# A Novel Protective MHC-I Haplotype Not Associated with Dominant Gag-Specific CD8^+^ T-Cell Responses in SIVmac239 Infection of Burmese Rhesus Macaques

**DOI:** 10.1371/journal.pone.0054300

**Published:** 2013-01-14

**Authors:** Naofumi Takahashi, Takushi Nomura, Yusuke Takahara, Hiroyuki Yamamoto, Teiichiro Shiino, Akiko Takeda, Makoto Inoue, Akihiro Iida, Hiroto Hara, Tsugumine Shu, Mamoru Hasegawa, Hiromi Sakawaki, Tomoyuki Miura, Tatsuhiko Igarashi, Yoshio Koyanagi, Taeko K. Naruse, Akinori Kimura, Tetsuro Matano

**Affiliations:** 1 AIDS Research Center, National Institute of Infectious Diseases, Tokyo, Japan; 2 The Institute of Medical Science, The University of Tokyo, Tokyo, Japan; 3 DNAVEC Corporation, Tsukuba, Japan; 4 Institute for Virus Research, Kyoto University, Kyoto, Japan; 5 Department of Molecular Pathogenesis, Medical Research Institute, Tokyo Medical and Dental University, Tokyo, Japan; University of California San Francisco, United States of America

## Abstract

Several major histocompatibility complex class I (MHC-I) alleles are associated with lower viral loads and slower disease progression in human immunodeficiency virus (HIV) and simian immunodeficiency virus (SIV) infections. Immune-correlates analyses in these MHC-I-related HIV/SIV controllers would lead to elucidation of the mechanism for viral control. Viral control associated with some protective MHC-I alleles is attributed to CD8^+^ T-cell responses targeting Gag epitopes. We have been trying to know the mechanism of SIV control in multiple groups of Burmese rhesus macaques sharing MHC-I genotypes at the haplotype level. Here, we found a protective MHC-I haplotype, *90-010-Id* (D), which is not associated with dominant Gag-specific CD8^+^ T-cell responses. Viral loads in five D^+^ animals became significantly lower than those in our previous cohorts after 6 months. Most D^+^ animals showed predominant Nef-specific but not Gag-specific CD8^+^ T-cell responses after SIV challenge. Further analyses suggested two Nef-epitope-specific CD8^+^ T-cell responses exerting strong suppressive pressure on SIV replication. Another set of five D^+^ animals that received a prophylactic vaccine using a Gag-expressing Sendai virus vector showed significantly reduced viral loads compared to unvaccinated D^+^ animals at 3 months, suggesting rapid SIV control by Gag-specific CD8^+^ T-cell responses in addition to Nef-specific ones. These results present a pattern of SIV control with involvement of non-Gag antigen-specific CD8^+^ T-cell responses.

## Introduction

Virus-specific CD8^+^ T-cell responses play a central role in the control of human immunodeficiency virus (HIV) and simian immunodeficiency virus (SIV) replication [1], [2], [3], [4], [5]. Genetic diversities of HLA or major histocompatibility complex class I (MHC-I) result in various patterns of CD8^+^ T-cell responses in HIV-infected individuals. Cumulative studies on HIV infection have indicated the association of MHC-I genotypes with higher or lower viral loads [6], [7], [8], [9], [10]. In some MHC-1 alleles associating with lower viral loads and slower disease progression, certain CD8^+^ T-cell responses restricted by these MHC-I molecules have been shown to be responsible for HIV control [11], [12], [13]. In rhesus macaque AIDS models, Mamu-A*01, Mamu-B*08, and Mamu-B*17 are known as protective alleles, and macaques possessing these alleles tend to show slower disease progression after SIVmac251/SIVmac239 challenge [14], [15], [16], [17].

Recent studies have indicated great contribution of CD8^+^ T-cell responses targeting Gag epitopes to reduction in viral loads in HIV/SIV infection [18], [19], [20], [21]. Viral control associated with some protective MHC-I alleles is attributed to Gag epitope-specific CD8^+^ T-cell responses [22], [23], [24]. For instance, CD8^+^ T-cell responses specific for the HLA-B*57-restricted Gag_240–249_ TW10 and HLA-B*27-restricted Gag_263–272_ KK10 epitopes exert strong suppressive pressure on HIV replication and frequently select for an escape mutation with viral fitness costs, leading to lower viral loads [22], [24], [25], [26], [27]. On the other hand, CD8^+^ T-cell responses targeting SIV antigens other than Gag, such as Mamu-B*08- or Mamu-B*17-restricted Vif and Nef epitopes, have been indicated to exert strong suppressive pressure on SIV replication [28], [29], [30], [31], [32], [33]. Accumulation of our knowledge on the potential of these non-Gag-specific as well as Gag-specific CD8^+^ T-cell responses for HIV/SIV control should be encouraged for elucidation of viral control mechanisms.

We have been examining SIVmac239 infection in multiple groups of Burmese rhesus macaques sharing MHC-I genotypes at the haplotype level and indicated an association of MHC-I haplotypes with AIDS progression [21], [34]. In our previous study, a group of macaques sharing MHC-I haplotype *90-120-Ia* (A) induced dominant Gag-specific CD8^+^ T-cell responses and tended to show slower disease progression after SIVmac239 challenge [21]. Prophylactic immunization of these A^+^ macaques with a DNA vaccine prime and a Gag-expressing Sendai virus (SeV-Gag) vector boost resulted in SIV control based on Gag-specific CD8^+^ T-cell responses [35], [36]. Accumulation of data on interaction between virus replication and T-cell responses in multiple groups of macaques sharing individual MHC-I haplotypes would provide great insights into our understanding of the mechanism for HIV/SIV control.

In the present study, we investigated SIVmac239 infection of a group of Burmese rhesus macaques possessing the MHC-I haplotype *90-010-Id* (D), which was not associated with dominant Gag-specific CD8^+^ T-cell responses. These animals had persistent viremia in the early phase but showed significant reduction of viral loads around 6 months after SIV challenge. Most D^+^ animals showed predominant Nef-specific but not Gag-specific CD8^+^ T-cell responses. This study presents a protective MHC-I haplotype, indicating the potential of non-Gag antigen-specific CD8^+^ T-cell responses to contribute to SIV control.

## Materials and Methods

### Ethics Statement

Animal experiments were carried out in National Institute of Biomedical Innovation (NIBP) and Institute for Virus Research in Kyoto University (IVRKU) after approval by the Committee on the Ethics of Animal Experiments of NIBP and IVRKU in accordance with the guidelines for animal experiments at NIBP, IVRKU, and National Institute of Infectious Diseases. To prevent viral transmission, animals were housed in individual cages allowing them to make sight and sound contact with one another, where the temperature was kept at 25°C with light in 12 hours per day. Animals were fed with apples and commercial monkey diet (Type CMK-2, Clea Japan, Inc. Tokyo). Blood collection, vaccination, and SIV challenge were performed under ketamine anesthesia. The endpoint for euthanasia was determined by typical signs of AIDS including reduction in peripheral CD4^+^ T-cell counts (less than 200 cells/µl), 10% loss of body weight, diarrhea, and general weakness. At euthanasia, animals were deeply anesthetized with pentobarbital under ketamine anesthesia, and then, whole blood was collected from left ventricle.

### Animal Experiments

We examined SIV infections in a group of Burmese rhesus macaques (n = 10) sharing the MHC-I haplotype *90-010-Id* (D). The determination of MHC-I haplotypes was based on the family study in combination with the reference strand-mediated conformation analysis (RSCA) of *Mamu-A* and *Mamu-B* genes and detection of major *Mamu-A* and *Mamu-B* alleles by cloning the reverse transcription (RT)-PCR products as described previously [21], [34], [37]. Macaques R01-012 and R01-009 used in our previous report [35] and macaques R03-021 and R03-016 used in an unpublished experiment were included in the present study. Five macaques R01-009, R06-020, R06-033, R03-021, and R03-016 received a prophylactic DNA prime/SeV-Gag boost vaccine (referred to as DNA/SeV-Gag vaccine) [35]. The DNA used for the vaccination, CMV-SHIVdEN, was constructed from an *env*-deleted and *nef*-deleted simian–human immunodeficiency virus SHIVMD14YE [38] molecular clone DNA (SIVGP1) and has the genes encoding SIVmac239 Gag, Pol, Vif, and Vpx, and HIV Tat and Rev. At the DNA vaccination, animals received 5 mg of CMV-SHIVdEN DNA intramuscularly. Six weeks after the DNA prime, animals received a single boost intranasally with 6×10^9^ cell infectious units (CIUs) of F-deleted replication-defective SeV-Gag [39], [40]. All animals were challenged intravenously with 1,000 TCID_50_ (50 percent tissue culture infective doses) of SIVmac239 [41]. At week 1 after SIV challenge, macaque R03-021 was inoculated with nonspecific immunoglobulin G (IgG) and macaques R03-016 with IgG purified from neutralizing antibody-positive plasma of chronically SIV-infected macaques in our previous experiment [42].

### Analysis of SIV Antigen-specific CD8^+^ T-cell Responses

SIV antigen-specific CD8^+^ T-cell responses were measured by flow-cytometric analysis of gamma interferon (IFN-γ) induction as described previously [43]. Autologous herpesvirus papio-immortalized B-lymphoblastoid cell lines (B-LCLs) were established from peripheral blood mononuclear cells (PBMCs) which were obtained from individual macaques before SIV challenge [44]. PBMCs obtained from SIV-infected macaques were cocultured with autologous B-LCLs pulsed with peptides or peptide pools using panels of overlapping peptides spanning the entire SIVmac239 Gag, Pol, Vif, Vpx, Vpr, Tat, Rev, Env, and Nef amino acid sequences. Alternatively, PBMCs were cocultured with B-LCLs infected with a vaccinia virus vector expressing SIVmac239 Gag for Gag-specific stimulation. Intracellular IFN-γ staining was performed using CytofixCytoperm kit (BD, Tokyo, Japan). Fluorescein isothiocianate-conjugated anti-human CD4 (BD), Peridinin chlorophyll protein (PerCP)-conjugated anti-human CD8 (BD), allophycocyanin Cy7 (APC-Cy7)-conjugated anti-human CD3 (BD), and phycoerythrin (PE)-conjugated anti-human IFN-γ antibodies (Biolegend, San Diego, CA) were used. Specific T-cell levels were calculated by subtracting non-specific IFN-γ^+^ T-cell frequencies from those after peptide-specific stimulation. Specific T-cell levels less than 100 cells per million PBMCs were considered negative.

### Sequencing Analysis of Plasma Viral Genomes

Viral RNAs were extracted using High Pure Viral RNA kit (Roche Diagnostics, Tokyo, Japan) from macaque plasma samples. Fragments of cDNAs encoding SIVmac239 Gag and Nef were amplified by nested RT-PCR from plasma RNAs and subjected to direct sequencing by using dye terminator chemistry and an automated DNA sequencer (Applied Biosystems, Tokyo, Japan) as described before [45]. Predominant non-synonymous mutations were determined.

### Statistical Analysis

Statistical analysis was performed using Prism software version 4.03 with significance levels set at a P value of <0.050 (GraphPad Software, Inc., San Diego, CA). Plasma viral loads were log transformed and compared by an unpaired two-tailed t test.

## Results

### Lower Viral Loads in D^+^ Macaques in the Chronic Phase of SIV Infection

**Figure 1:**
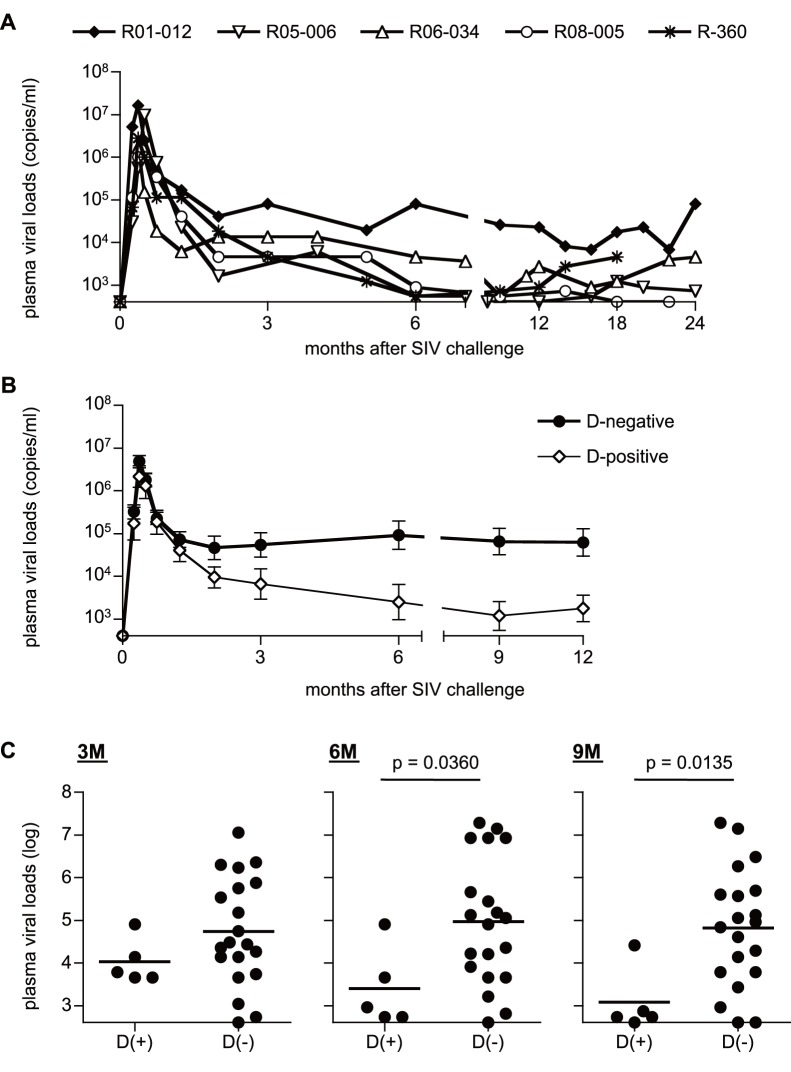
Plasma viral loads after SIVmac239 challenge in unvaccinated macaques. Plasma viral loads (SIV *gag* RNA copies/ml plasma) were determined as described previously [35]. The lower limit of detection is approximately 4×10^2^ copies/ml. (A) Changes in plasma viral loads after challenge in unvaccinated macaques possessing MHC-I haplotype D. (B) Changes in geometric means of plasma viral loads after challenge in five unvaccinated D^+^ animals in the present study and twenty D^−^ animals in our previous cohorts [21]. Three of twenty D^−^ animals were euthanized because of AIDS before 12 months, and we compared viral loads between D^+^ and D^−^ animals until 12 months. (C) Comparison of plasma viral loads at 3 months (left panel), 6 months (middle panel), and 9 months (right panel) between the unvaccinated D^+^ and the D^−^ animals. Viral loads at 6 months and 9 months in D^+^ animals were significantly lower than those in the latter D^−^ animals (p = 0.0360 at 6 months and p = 0.0135 at 9 months by t-test).

We first investigated SIVmac239 infection of five unvaccinated Burmese rhesus macaques sharing the MHC-I haplotype D (referred to as D^+^ macaques). Confirmed MHC-I alleles consisting of this haplotype is Mamu-A1*032:02, Mamu-B*004:01, and Mamu-B*102:01:01. These animals showed lower set-point plasma viral loads (Fig. 1). Comparison of plasma viral loads between these five animals and our previous cohorts of SIVmac239-infected Burmese D-negative (D^−^) rhesus macaques (n = 20) [21] revealed no significant difference at 3 months after SIV challenge (p = 0.2436 by t-test), but viral loads in the former D^+^ animals became significantly lower than the latter after 6 months (p = 0.0360 at 6 months and p = 0.0135 at 9 months by t-test; Fig. 1). Four of these five macaques sharing MHC-I haplotype D showed low viral loads, less than 5×10^3^ copies/ml, after 6 months, whereas macaque R01-012 maintained relatively higher viral loads.

### Predominant Nef-specific CD8^+^ T-cell Responses

**Figure 2:**
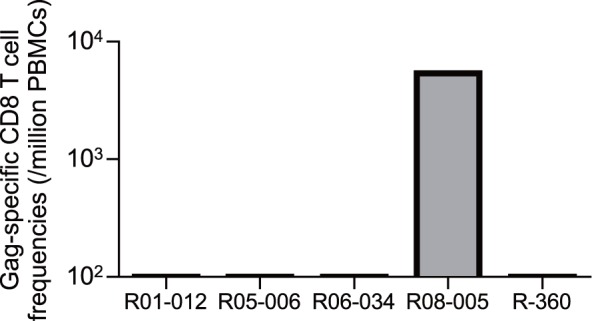
SIV Gag-specific CD8^+^ T-cell responses in unvaccinated D^+^ macaques at week 2 after SIVmac239 challenge.

We examined SIV antigen-specific CD8^+^ T-cell responses by detection of antigen-specific IFN-γ induction. In the very acute phase, we did not have enough PBMC samples for measurement of individual SIV antigen-specific CD8^+^ T-cell responses and focused on examining Gag-specific CD8^+^ T-cell responses in most animals. At week 2 after challenge, Gag-specific CD8^+^ T-cell responses were undetectable in four of five animals (Fig. 2).

**Figure 3:**
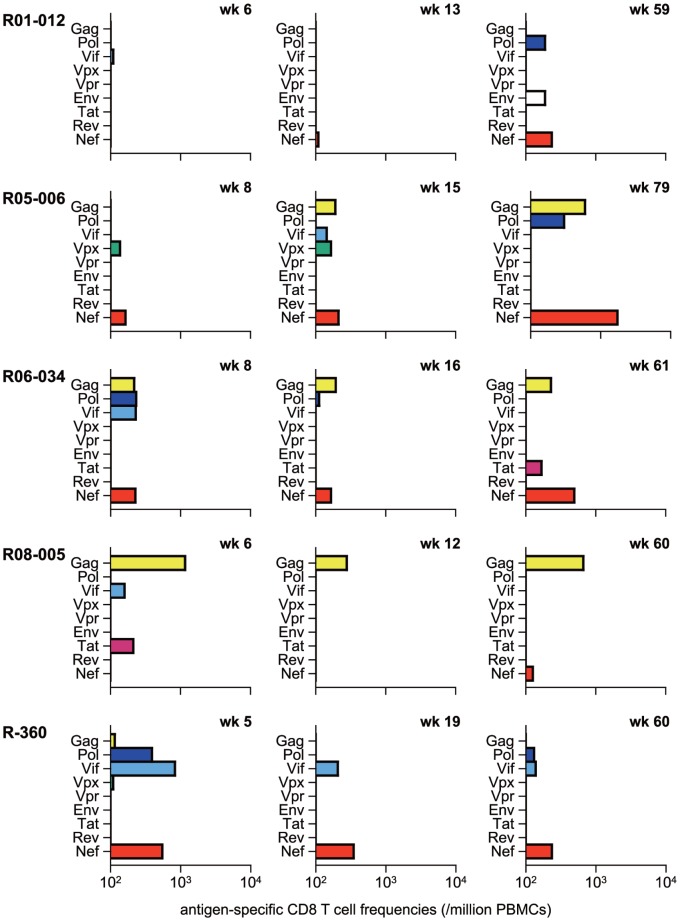
SIV antigen-specific CD8^+^ T-cell responses in unvaccinated D^+^ macaques. Responses were measured by the detection of antigen-specific IFN-γ induction in PBMCs obtained at indicated time points after SIVmac239 challenge.

We then examined CD8^+^ T-cell responses specific for individual SIV antigens in the early and the late phases (Fig. 3). Nef-specific but not Gag-specific CD8^+^ T-cell responses were predominant in most D^+^ animals. Gag-specific CD8^+^ T-cell responses were dominantly induced in macaque R08-005 showing very low set-point viral loads. Macaque R01-012 having higher viral loads showed poor CD8^+^ T-cell responses in the early phase.

**Figure 4:**
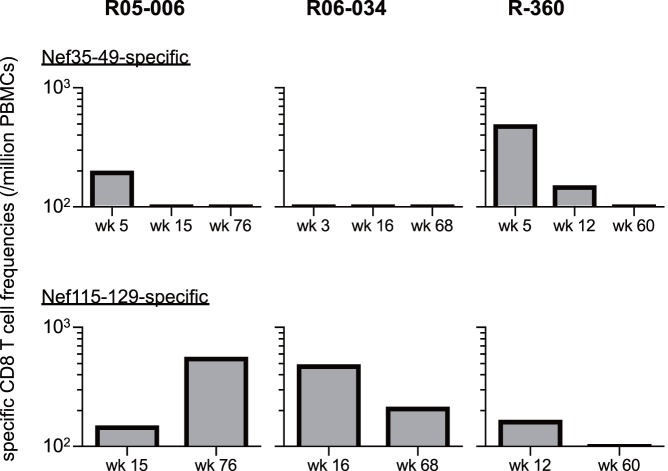
SIV Nef-specific CD8^+^ T-cell responses in macaques R05-006, R06-034, and R-360. Nef_35–49_-specific (upper panels) and Nef_115–129_-specific (lower panels) CD8^+^ T-cell responses were examined at indicated time points after SIVmac239 challenge.

Among four D^+^ animals controlling SIV replication with less than 5×10^3^ copies/ml of plasma viral loads after 6 months, Gag-specific CD8^+^ T-cell responses were dominant only in macaque R08-005, while efficient Nef-specific CD8^+^ T-cell responses were induced in the remaining three, suggesting possible contribution of Nef-specific CD8^+^ T-cell responses to SIV control in these three controllers (R05-006, R06-034, and R-360). We then attempted to localize Nef CD8^+^ T-cell epitopes shared in these animals and found Nef_35–49_-specific and Nef_115–129_-specific CD8^+^ T-cell responses (Fig. 4), although we did not have enough samples for mapping the exact epitopes.

### Reduction of Viral Loads in the Early Phase of SIV Infection by Prophylactic Vaccination

**Figure 5:**
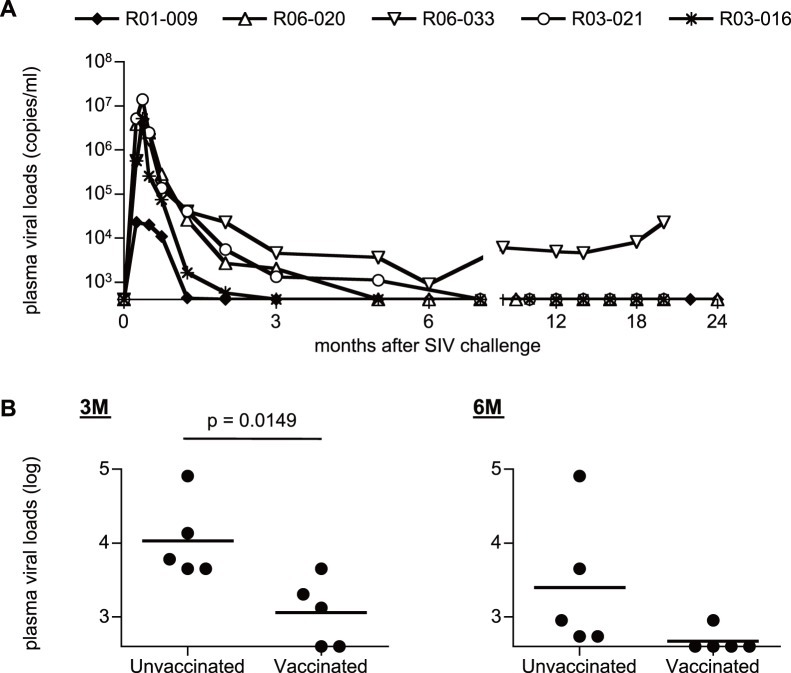
Plasma viral loads after SIVmac239 challenge in vaccinated D^+^ macaques. (A) Changes in plasma viral loads after challenge vaccinated macaques possessing MHC-I haplotype D. (B) Comparison of plasma viral loads at 3 months (left panel) and 6 months (right panel) between five unvaccinated D^+^ and five vaccinated D^+^ animals. Viral loads at 3 months in vaccinated animals were significantly lower than those in the unvaccinated (p = 0.0149 by t-test).

We also investigated SIVmac239 infection of additional five, vaccinated Burmese rhesus macaques sharing the MHC-I haplotype D. These animals received a prophylactic DNA/SeV-Gag vaccination. In four of these five vaccinated macaques, plasma viremia became undetectable after 6 months, while macaque R06-033 showed persistent viremia (Fig. 5A). Difference in viral loads between unvaccinated and vaccinated D^+^ animals was unclear in the acute phase, but the latter vaccinees showed significant reduction in viral loads compared to those in the former unvaccinated at 3 months (p = 0.0360; Fig. 5B). After 6 months, unvaccinated animals also showed reduced viral loads, and the difference in viral loads between unvaccinated and vaccinated became unclear.

**Figure 6:**
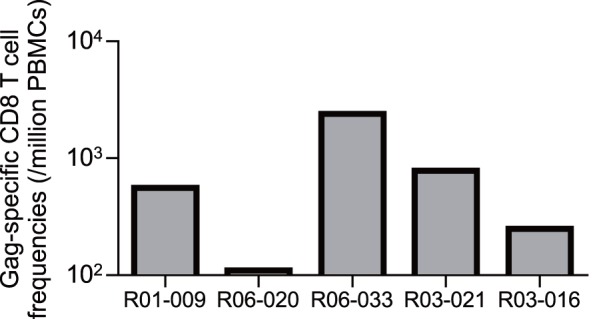
SIV Gag-specific CD8^+^ T-cell responses in vaccinated D^+^ macaques at week 2 after SIVmac239 challenge.

**Figure 7:**
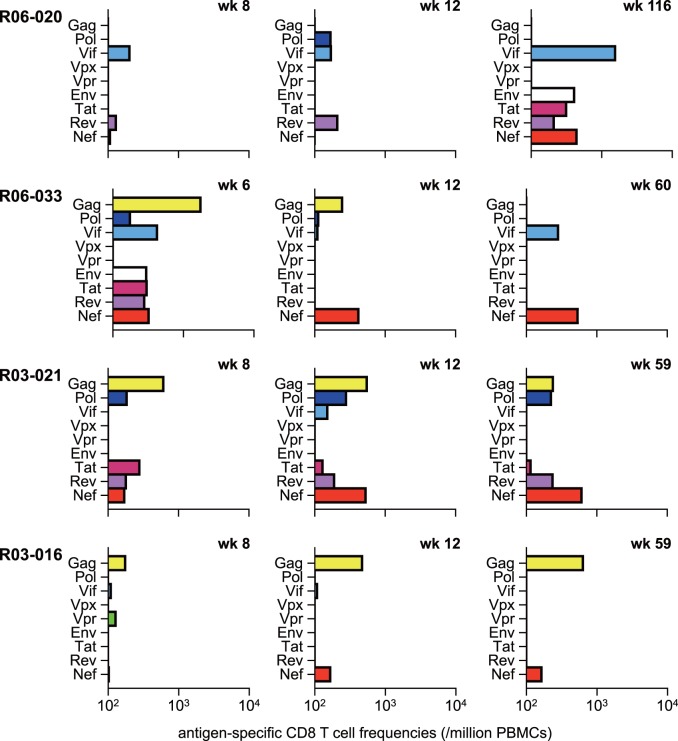
SIV antigen-specific CD8^+^ T-cell responses in vaccinated D^+^ animals after SIVmac239 challenge. Samples for this analysis were unavailable in macaque R01-009.

In contrast to unvaccinated D^+^ animals, all five vaccinated animals elicited Gag-specific CD8^+^ T-cell responses at week 2 after challenge (Fig. 6), reflecting the effect of prophylactic vaccination. We then examined CD8^+^ T-cell responses specific for individual SIV antigens in these vaccinated animals (Fig. 7). Samples for this analysis were unavailable in vaccinated macaque R01-009. Vaccinated animals except for macaque R06-020 showed dominant Gag-specific CD8^+^ T-cell responses even at 1–2 months. However, Gag-specific CD8^+^ T-cell responses became not dominant after 1 year, while Nef-specific or Vif-specific CD8^+^ T-cell responses became predominant, instead, in most vaccinees except for macaque R03-016.

**Figure 8:**
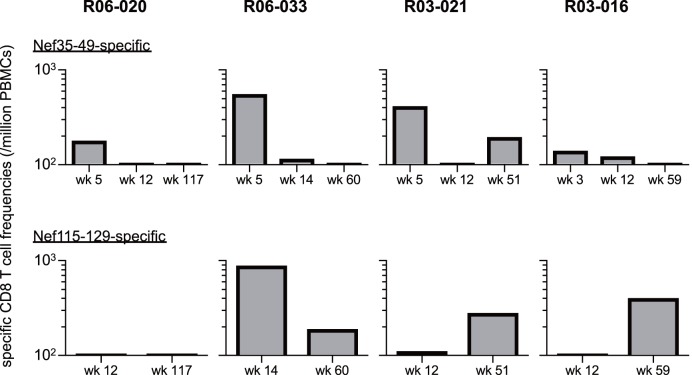
SIV Nef-specific CD8^+^ T-cell responses in macaques R06-020, R06-033, R03-021, and R03-016. Nef_35–49_-specific (upper panels) and Nef_115–129_-specific (lower panels) CD8^+^ T-cell responses were examined at indicated time points after SIVmac239 challenge.

Like three unvaccinated macaques (R05-006, R06-034, and R-360), vaccinated D^+^ animals induced Nef_35–49_-specific and Nef_115–129_-specific CD8^+^ T-cell responses after SIV challenge (Fig. 8). In analyses of three unvaccinated (Fig. 4) and four vaccinated animals (Fig. 8), Nef_35–49_-specific CD8^+^ T-cell responses were induced in the early phase in six animals but mostly became undetectable in the chronic phase. Nef_115–129_-specific CD8^+^ T-cell responses were also induced in most animals except for macaque R06-020 which showed Nef_112–126_-specific ones in the chronic phase (data not shown). Macaques R05-006, R03-021, and R03-016 showed efficient Nef_115–129_-specific CD8^+^ T-cell responses not in the early phase but in the chronic phase. In contrast, vaccinated animal R06-033 that failed to control viremia showed higher Nef_115–129_-specific CD8^+^ T-cell responses in the early phase than those in the chronic phase.

### Selection of Mutations in Nef CD8^+^ T-cell Epitope-coding Regions

**Figure 9:**
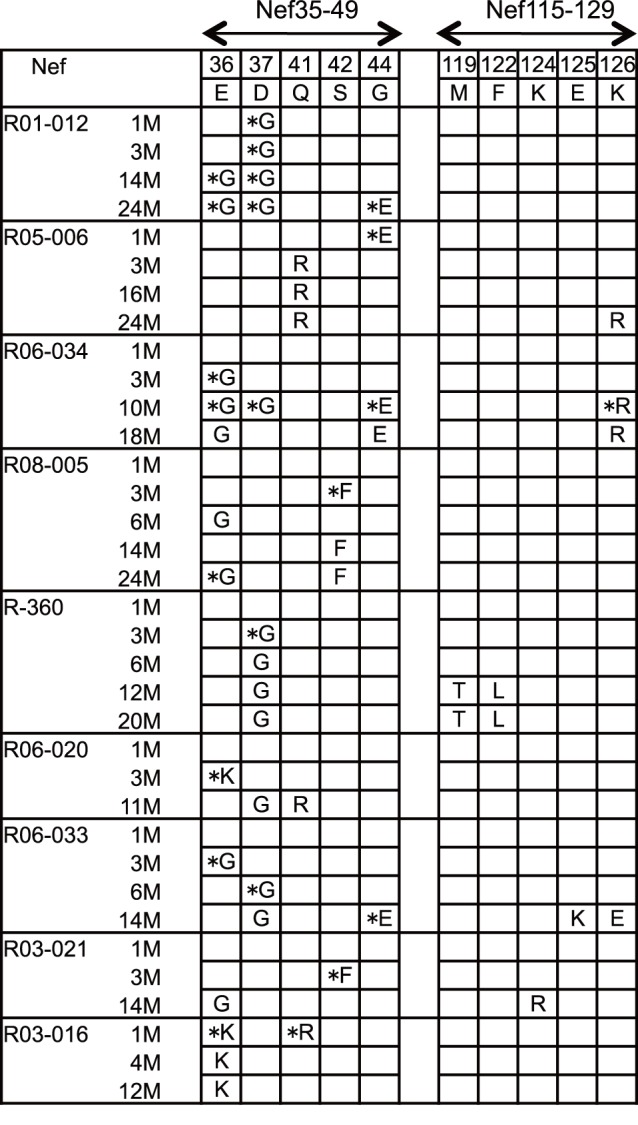
Predominant non-synonymous mutations in Nef_35–49_-coding and Nef_115–129_-coding regions of viral cDNAs in D^+^ animals after SIVmac239 challenge. Amino acid substitutions are shown. Detection of similar levels of wild-type and mutant sequences at the residue is indicated by asterisks. Samples for this analysis were unavailable in macaque R01-009.

To see the effect of selective pressure by Nef-specific CD8^+^ T-cell responses on viral genome mutations, we next analyzed nucleotide sequences in viral *nef* cDNAs amplified from plasma RNAs obtained at several time points after SIV challenge. Nonsynonymous mutations detected predominantly in Nef_35–49_-coding and Nef_115–129_-coding regions were as shown in Fig. 9. Remarkably, all the unvaccinated and vaccinated D^+^ animals showed rapid selection of mutations in the Nef_35–49_-coding region in 3 months. On the other hand, mutations in the Nef_115–129_-coding region were observed in the late phase in all the three unvaccinated animals eliciting Nef_115–129_-specific CD8^+^ T-cell responses. These mutations were also detected in two of three vaccinated animals eliciting Nef_115–129_-specific CD8^+^ T-cell responses.

We also analyzed viral *gag* sequences to see the effect of Gag-specific CD8^+^ T-cell pressure on viral genome mutations in vaccinated animals (data not shown). Our previous study [35] showed rapid selection of a mutation leading to a glutamine (Q)-to-lysine (K) change at the 58th residue in Gag (Q58K) at week 5 in vaccinated macaque R01-009, although no more samples were available for this sequencing analysis. This Q58K mutation results in escape from Gag_50–65_-specific CD8^+^ T-cell recognition. In the present study, macaque R03-016 showed rapid selection of a mutation leading to a K-to-asparagine (N) change at the 478th residue in Gag in 1 month. These results may reflect rapid disappearance of detectable plasma viremia in 1 or 2 months in these two vaccinees. Macaque R06-020 showed selection of a *gag* mutation in 3 months, while other two vaccinees (R06-033 and R03-021) selected no *gag* mutation in the early phase.

## Discussion

HIV infection in humans with polymorphic MHC-I genotypes induces various patterns of viral antigen-specific CD8^+^ T-cell responses. Previous studies have found several protective MHC-I alleles associated with lower viral loads and slower disease progression in HIV/SIV infection [7], [13], [14], [16], [17]. Elucidation of the mechanisms of viral control associated with individual protective MHC-I alleles would contribute to HIV cure and vaccine-based prevention. Because CD8^+^ T-cell responses specific for some MHC-I-restricted epitopes can be affected by those specific for other MHC-I-restricted epitopes due to immunodominance [29], [46], [47], macaque groups sharing MHC-I genotypes at the haplotype level are useful for the analysis of cooperation of multiple epitope-specific CD8^+^ T-cell responses. Previously, we reported a group of Burmese rhesus macaques sharing MHC-I haplotype *90-120-Ia* (A), which dominantly induce Gag-specific CD8^+^ T-cell responses and tend to show slower disease progression after SIVmac239 challenge [21]. In the present study, we presented another type of protective MHC-I haplotype, which is not associated with dominant Gag-specific CD8^+^ T-cell responses. Significant reduction of viral loads in unvaccinated macaques possessing this D haplotype compared to those in D^−^ macaques was observed after 6 months. Analysis of SIV infection in macaques sharing this protective MHC-I haplotype would lead to understanding of CD8^+^ T-cell cooperation for viral control.

Analyses of antigen-specific CD8^+^ T-cell responses after SIVmac239 challenge indicate that this MHC-I haplotype D is associated with predominant Nef-specific CD8^+^ T-cell responses. Nef-specific CD8^+^ T-cell responses were efficiently induced in all SIV controllers, whereas Gag-specific CD8^+^ T-cell responses were dominant in only one of them. We found Nef_35–49_-specific and Nef_115–129_-specific CD8^+^ T-cell responses shared in D^+^ animals. We were unable to determine the MHC-I alleles restricting these epitopes, but these responses are not usually induced in our previous D^−^ cohorts and considered to be associated with this MHC-I haplotype D.

Sequencing analysis of viral genomes showed rapid selection of mutations in the Nef_36–44_-coding region within 3 months in all the D^+^ animals. This is consistent with our results that Nef_35–49_-specific CD8^+^ T-cell responses were mostly induced in the early phase but undetectable in the chronic phase. These mutations were not consistently selected in our previous D^−^ cohorts and thus considered as MHC-I haplotype D-associated mutations. This suggests strong selective pressure by Nef_35–49_-specific CD8^+^ T-cell responses in the acute phase of SIVmac239 infection in D^+^ macaques, although it remains undetermined whether these mutations result in viral escape from Nef_35–49_-specific CD8^+^ T-cell recognition.

**Figure 10:**
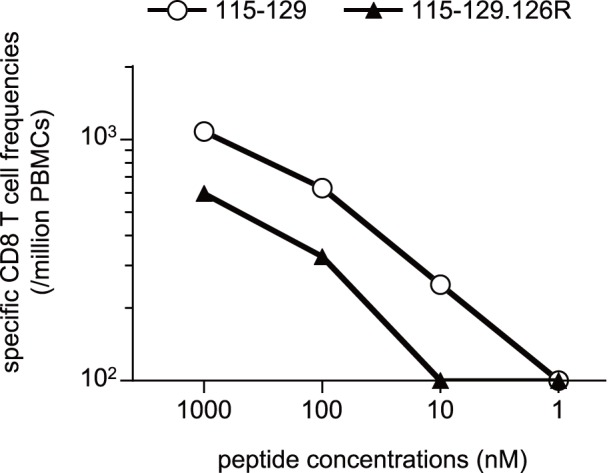
IFN-γ induction in CD8^+^ T cells after stimulation with the wild-type or the mutant peptide. PBMCs obtained at week 31 from macaque R06-033 were stimulated by coculture with B-LCL pulsed with indicated concentrations of the wild-type Nef_115–129_ peptide (open circles, 115–129, LAIDMSHFIKEKGGL) or the mutant Nef_115–129_ peptide with a K126R alteration (closed triangles, 115–129.126R, LAIDMSHFIKERGGL).

Nef_115–129_-specific CD8^+^ T-cell responses were detected in six D^+^ animals. In five of them, nonsynonymous mutations in the Nef_119–126_-coding region were observed in the chronic phase. At least, we confirmed viral escape from Nef_115–129_-specific CD8^+^ T-cell recognition by a mutation leading to a K-to-arginine (R) (K126R) substitution at Nef residue 126 (Fig. 10). The number of nonsynonymous substitutions per the number of sites estimated to be nonsynonymous (dN) exceeded that estimated to be synonymous (dS) during the evolution process of Nef_115–129_-coding region, but the value did not show statistically significant difference from that of neutral selection. Among three unvaccinated animals that controlled SIV replication without dominant Gag-specific CD8^+^ T-cell responses, amino acid substitutions in the Nef_119–126_-coding region were observed in a year in macaques R06-034 and R-360 but after 2 years in macaque R05-006. The former two animals tended to show earlier increases in plasma viral loads in the chronic phase, while the latter R05-006 maintained higher frequencies of Nef_115–129_-specific CD8^+^ T-cell responses. Nef_115–129_-specific CD8^+^ T-cell responses were efficient in the chronic phase in vaccinated controllers R03-021 and R03-016 but decreased in R06-033 that failed to contain SIV replication. Although a possible effect of this haplotype-associated factors other than CD8^+^ T-cell responses such as NK activity on SIV infection [48], [49], [50] remains undetermined, these results imply involvement of Nef-specific CD8^+^ T-cell responses in the SIV control associated with MHC-I haplotype D.

Unvaccinated macaque R08-005 dominantly elicited Gag antigen-specific CD8^+^ T-cell responses and showed rapid selection of a mutation encoding Gag 257 residue, which was not observed in any other D^+^ animals. Nef-specific CD8^+^ T-cell responses were detectable only at week 2 in the acute phase (data not shown) and a mutation in the Nef_42_-coding region was rapidly selected. It is speculated that those dominant Gag-specific CD8^+^ T-cell responses associated with the second, non-D MHC-I haplotype were effective in this animal. Nef_35–49_-specific CD8^+^ T-cell responses may not be efficient due to immunodominance but exert some suppressive pressure on viral replication.

DNA/SeV-Gag vaccination resulted in earlier reduction of viral loads after SIV challenge. Vaccinees showed significantly lower viral loads at 3 months than those in unvaccinated animals. Gag-specific CD8^+^ T-cell responses were elicited at week 2 in all the vaccinees but not in the unvaccinated except for one animal R08-005. No *gag* mutations were shared in the vaccinees in the acute phase, but three of them showed rapid selection of individual non-synonymous mutations in *gag*. Rapid selection of mutations in the Nef_36–44_-coding region was consistently detected even in these vaccinees. These results suggest broader CD8^+^ T-cell responses consisting of dominant vaccine antigen Gag-specific and inefficient naive-derived Nef-specific ones in the acute phase. In three vaccinated animals, Gag-specific CD8^+^ T-cell responses became lower or undetectable, and instead, Nef-specific CD8^+^ T-cell responses became predominant in the chronic phase.

In summary, we found a protective MHC-I haplotype not associated with dominant Gag-specific CD8^+^ T-cell responses in SIVmac239 infection. Our results in D^+^ macaques suggest suppressive pressure by Nef_35–49_-specific and Nef_115–129_-specific CD8^+^ T-cell responses on SIV replication, contributing to reduction in set-point viral loads. DNA/SeV-Gag-vaccinated D^+^ animals induced Gag-specific CD8^+^ T-cell responses in addition to Nef-specific ones after SIV challenge, resulting in earlier containment of SIV replication. This study presents a pattern of SIV control with involvement of non-Gag antigen-specific CD8^+^ T-cell responses, contributing to accumulation of our knowledge on HIV/SIV control mechanisms.

## References

[1] Borrow P, Lewicki H, Hahn BH, Shaw GM, Oldstone MB (1994) Virus-specific CD8+ cytotoxic T-lymphocyte activity associated with control of viremia in primary human immunodeficiency virus type 1 infection. J Virol 68: 6103–6110. 8057491 10.1128/jvi.68.9.6103-6110.1994 PMC237022

[2] Koup RA, Safrit JT, Cao Y, Andrews CA, McLeod G, et al (1994) Temporal association of cellular immune responses with the initial control of viremia in primary human immunodeficiency virus type 1 syndrome. J Virol 68: 4650–4655. 8207839 10.1128/jvi.68.7.4650-4655.1994 PMC236393

[3] Matano T, Shibata R, Siemon C, Connors M, Lane HC, et al (1998) Administration of an anti-CD8 monoclonal antibody interferes with the clearance of chimeric simian/human immunodeficiency virus during primary infections of rhesus macaques. J Virol 72: 164–169. 9420212 10.1128/jvi.72.1.164-169.1998 PMC109361

[4] Jin X, Bauer DE, Tuttleton SE, Lewin S, Gettie A, et al (1999) Dramatic rise in plasma viremia after CD8+ T cell depletion in simian immunodeficiency virus-infected macaques. J Exp Med 189: 991–998. 10075982 10.1084/jem.189.6.991 PMC2193038

[5] Schmitz JE, Kuroda MJ, Santra S, Sasseville VG, Simon MA, et al (1999) Control of viremia in simian immunodeficiency virus infection by CD8+ lymphocytes. Science 283: 857–860. 9933172 10.1126/science.283.5403.857

[6] Carrington M, Nelson GW, Martin MP, Kissner T, Vlahov D, et al (1999) HLA and HIV-1: heterozygote advantage and B*35-Cw*04 disadvantage. Science 283: 1748–1752. 10073943 10.1126/science.283.5408.1748

[7] Migueles SA, Sabbaghian MS, Shupert WL, Bettinotti MP, Marincola FM, et al (2000) HLA B*5701 is highly associated with restriction of virus replication in a subgroup of HIV-infected long term nonprogressors. Proc Natl Acad Sci USA 97: 2709–2714. 10694578 10.1073/pnas.050567397 PMC15994

[8] Tang J, Tang S, Lobashevsky E, Myracle AD, Fideli U, et al (2002) Favorable and unfavorable HLA class I alleles and haplotypes in Zambians predominantly infected with clade C human im- munodeficiency virus type 1. J Virol 76: 8276–8284. 12134033 10.1128/JVI.76.16.8276-8284.2002 PMC155130

[9] Kiepiela P, Leslie AJ, Honeyborne I, Ramduth D, Thobakgale C, et al (2004) Dominant influence of HLA-B in mediating the potential co-evolution of HIV and HLA. Nature 432: 769–775. 15592417 10.1038/nature03113

[10] Leslie A, Matthews PC, Listgarten J, Carlson JM, Kadie C, et al (2010) Additive contribution of HLA class I alleles in the immune control of HIV-1 infection. J Virol 84: 9879–9888. 20660184 10.1128/JVI.00320-10 PMC2937780

[11] Altfeld M, Addo MM, Rosenberg ES, Hecht FM, Lee PK, et al (2003) Influence of HLA-B57 on clinical presentation and viral control during acute HIV-1 infection. AIDS 17: 2581–2591. 14685052 10.1097/00002030-200312050-00005

[12] Altfeld M, Kalife ET, Qi Y, Streeck H, Lichterfeld M, et al (2006) HLA alleles associated with delayed progression to AIDS contribute strongly to the initial CD8+ T cell response against HIV-1. PLoS Med 3: e403. 17076553 10.1371/journal.pmed.0030403 PMC1626551

[13] Goulder PJ, Watkins DI (2008) Impact of MHC class I diversity on immune control of immunodeficiency virus replication. Nat Rev Immunol 8: 619–630. 18617886 10.1038/nri2357 PMC2963026

[14] Muhl T, Krawczak M, Ten Haaft P, Hunsmann G, Sauermann U (2002) MHC class I alleles influence set-point viral load and survival time in simian immunodeficiency virus-infected rhesus monkeys. J Immunol 169: 3438–3446. 12218167 10.4049/jimmunol.169.6.3438

[15] Mothe BR, Weinfurter J, Wang C, Rehrauer W, Wilson N, et al (2003) Expression of the major histocompatibility complex class I molecule Mamu-A*01 is associated with control of simian immunodeficiency virus SIVmac239 replication. J Virol 77: 2736–2740. 12552014 10.1128/JVI.77.4.2736-2740.2003 PMC141082

[16] Yant LJ, Friedrich TC, Johnson RC, May GE, Maness NJ, et al (2006) The high-frequency major histocompatibility complex class I allele Mamu-B*17 is associated with control of simian immunodeficiency virus SIVmac239 replication. J Virol 80: 5074–5077. 16641299 10.1128/JVI.80.10.5074-5077.2006 PMC1472056

[17] Loffredo JT, Maxwell J, Qi Y, Glidden CE, Borchardt GJ, et al (2007) Mamu-B*08-positive macaques control simian immunodeficiency virus replication. J Virol 81: 8827–8832. 17537848 10.1128/JVI.00895-07 PMC1951344

[18] Edwards BH, Bansal A, Sabbaj S, Bakari J, Mulligan MJ, et al (2002) Magnitude of functional CD8+ T-cell responses to the gag protein of human immunodeficiency virus type 1 correlates inversely with viral load in plasma. J Virol 76: 2298–2305. 11836408 10.1128/jvi.76.5.2298-2305.2002 PMC135950

[19] Zuniga R, Lucchetti A, Galvan P, Sanchez S, Sanchez C, et al (2006) Relative dominance of Gag p24-specific cytotoxic T lymphocytes is associated with human immunodeficiency virus control. J Virol 80: 3122–3125. 16501126 10.1128/JVI.80.6.3122-3125.2006 PMC1395458

[20] Kiepiela P, Ngumbela K, Thobakgale C, Ramduth D, Honeyborne I, et al (2007) CD8+ T-cell responses to different HIV proteins have discordant associations with viral load. Nat Med 13: 46–53. 17173051 10.1038/nm1520

[21] Nomura T, Yamamoto H, Shiino T, Takahashi N, Nakane T, et al (2012) Association of major histocompatibility complex class I haplotypes with disease progression after simian immunodeficiency virus challenge in Burmese rhesus macaques. J Virol 86: 6481–6490. 22491464 10.1128/JVI.07077-11 PMC3393527

[22] Schneidewind A, Brockman MA, Yang R, Adam RI, Li B, et al (2007) Escape from the dominant HLA-B27-restricted cytotoxic T-lymphocyte response in Gag is associated with a dramatic reduction in human immunodeficiency virus type 1 replication. J Virol 81: 12382–12393. 17804494 10.1128/JVI.01543-07 PMC2169010

[23] Emu B, Sinclair E, Hatano H, Ferre A, Shacklett B, et al (2008) HLA class I-restricted T-cell responses may contribute to the control of human immunodeficiency virus infection, but such responses are not always necessary for long-term virus control. J Virol 82: 5398–5407. 18353945 10.1128/JVI.02176-07 PMC2395228

[24] Miura T, Brockman MA, Schneidewind A, Lobritz M, Pereyra F, et al (2009) HLA-B57/B*5801 human immunodeficiency virus type 1 elite controllers select for rare gag variants associated with reduced viral replication capacity and strong cytotoxic T-lymphocyte recognition. J Virol 83: 2743–2755. 19116253 10.1128/JVI.02265-08 PMC2648254

[25] Leslie AJ, Pfafferott KJ, Chetty P, Draenert R, Addo MM, et al (2004) HIV evolution: CTL escape mutation and reversion after transmission. Nat Med 10: 282–289. 14770175 10.1038/nm992

[26] Martinez-Picado J, Prado JG, Fry EE, Pfafferott K, Leslie A, et al (2006) Fitness cost of escape mutations in p24 Gag in association with control of human immunodeficiency virus type 1. J Virol 80: 3617–3623. 16537629 10.1128/JVI.80.7.3617-3623.2006 PMC1440414

[27] Crawford H, Prado JG, Leslie A, Hue S, Honeyborne I, et al (2007) Compensatory mutation partially restores fitness and delays reversion of escape mutation within the immunodominant HLA-B*5703-restricted Gag epitope in chronic human immunodeficiency virus type 1 infection. J Virol 81: 8346–8351. 17507468 10.1128/JVI.00465-07 PMC1951305

[28] Friedrich TC, Valentine LE, Yant LJ, Rakasz EG, Piaskowski SM, et al (2007) Subdominant CD8+ T-cell responses are involved in durable control of AIDS virus replication. J Virol 81: 3465–3476. 17251286 10.1128/JVI.02392-06 PMC1866056

[29] Loffredo JT, Bean AT, Beal DR, Leon EJ, May GE, et al (2008) Patterns of CD8+ immunodominance may influence the ability of Mamu-B*08-positive macaques to naturally control simian immunodeficiency virus SIVmac239 replication. J Virol 82: 1723–1738. 18057253 10.1128/JVI.02084-07 PMC2258706

[30] Maness NJ, Yant LJ, Chung C, Loffredo JT, Friedrich TC, et al (2008) Comprehensive immunological evaluation reveals surprisingly few differences between elite controller and progressor Mamu-B*17-positive simian immunodeficiency virus-infected rhesus macaques. J Virol 82: 5245–5254. 18385251 10.1128/JVI.00292-08 PMC2395202

[31] Valentine LE, Loffredo JT, Bean AT, Leon EJ, MacNair CE, et al (2009) Infection with “escaped” virus variants impairs control of simian immunodeficiency virus SIVmac239 replication in Mamu-B*08-positive macaques. J Virol 83: 11514–11527. 19726517 10.1128/JVI.01298-09 PMC2772717

[32] Budde ML, Greene JM, Chin EN, Ericsen AJ, Scarlotta M, et al (2012) Specific CD8+ T cell responses correlate with control of simian immunodeficiency virus replication in Mauritian cynomolgus macaques. J Virol 86: 7596–7604. 22573864 10.1128/JVI.00716-12 PMC3416303

[33] Mudd PA, Martins MA, Ericsen AJ, Tully DC, Power KA, et al (2012) Vaccine-induced CD8+ T cells control AIDS virus replication. Nature 491: 129–133. 23023123 10.1038/nature11443 PMC3883109

[34] Naruse TK, Chen Z, Yanagida R, Yamashita T, Saito Y, et al (2010) Diversity of MHC class I genes in Burmese-origin rhesus macaques. Immunogenetics 62: 601–611. 20640416 10.1007/s00251-010-0462-z

[35] Matano T, Kobayashi M, Igarashi H, Takeda A, Nakamura H, et al (2004) Cytotoxic T lymphocyte-based control of simian immunodeficiency virus replication in a preclinical AIDS vaccine trial. J Exp Med 199: 1709–1718. 15210746 10.1084/jem.20040432 PMC2212812

[36] Kawada M, Tsukamoto T, Yamamoto H, Iwamoto N, Kurihara K, et al (2008) Gag-specific cytotoxic T-lymphocyte-based control of primary simian immunodeficiency virus replication in a vaccine trial. J Virol 82: 10199–10206. 18667518 10.1128/JVI.01103-08 PMC2566295

[37] Tanaka-Takahashi Y, Yasunami M, Naruse T, Hinohara K, Matano T, et al (2007) Reference strand-mediated conformation analysis-based typing of multiple alleles in the rhesus macaque MHC class I Mamu-A and Mamu-B loci. Electrophoresis 28: 918–924. 17309048 10.1002/elps.200600586

[38] Shibata R, Maldarelli F, Siemon C, Matano T, Parta M, et al (1997) Infection and pathogenicity of chimeric simian-human immunodeficiency viruses in macaques: determinants of high virus loads and CD4 cell killing. J Infect Dis 176: 362–373. 9237701 10.1086/514053

[39] Li HO, Zhu YF, Asakawa M, Kuma H, Hirata T, et al (2000) A cytoplasmic RNA vector derived from nontransmissible Sendai virus with efficient gene transfer and expression. J Virol 74: 6564–6569. 10864670 10.1128/jvi.74.14.6564-6569.2000 PMC112166

[40] Takeda A, Igarashi H, Nakamura H, Kano M, Iida A, et al (2003) Protective efficacy of an AIDS vaccine, a single DNA priming followed by a single booster with a recombinant replication-defective Sendai virus vector, in a macaque AIDS model. J Virol 77: 9710–9715. 12915583 10.1128/JVI.77.17.9710-9715.2003 PMC187428

[41] Kestler HW III, Ringler DJ, Mori K, Panicali DL, Sehgal PK, et al (1991) Importance of the nef gene for maintenance of high virus loads and for development of AIDS. Cell 65: 651–662. 2032289 10.1016/0092-8674(91)90097-i

[42] Yamamoto H, Kawada M, Takeda A, Igarashi H, Matano T (2007) Post-infection immunodeficiency virus control by neutralizing antibodies. PLoS One 2: e540. 17579714 10.1371/journal.pone.0000540 PMC1890307

[43] Iwamoto N, Tsukamoto T, Kawada M, Takeda A, Yamamoto H, et al (2010) Broadening of CD8+ cell responses in vaccine-based simian immunodeficiency virus controllers. AIDS 24: 2777–2787. 21045637 10.1097/QAD.0b013e3283402206

[44] Voss G, Nick S, Stahl-Hennig C, Ritter K, Hunsmann G (1992) Generation of macaque B lymphoblastoid cell lines with simian Epstein-Barr-like viruses: transformation procedure, characterization of the cell lines and occurrence of simian foamy virus. J Virol Methods 39: 185–195. 1331148 10.1016/0166-0934(92)90137-3

[45] Kawada M, Igarashi H, Takeda A, Tsukamoto T, Yamamoto H, et al (2006) Involvement of multiple epitope-specific cytotoxic T-lymphocyte responses in vaccine-based control of simian immunodeficiency virus replication in rhesus macaques. J Virol 80: 1949–1958. 16439550 10.1128/JVI.80.4.1949-1958.2006 PMC1367167

[46] Tenzer S, Wee E, Burgevin A, Stewart-Jones G, Friis L, et al (2009) Antigen processing influences HIV-specific cytotoxic T lymphocyte immunodominance. Nat Immunol 10: 636–646. 19412183 10.1038/ni.1728

[47] Ishii H, Kawada M, Tsukamoto T, Yamamoto H, Matsuoka S, et al (2012) Impact of vaccination on cytotoxic T lymphocyte immunodominance and cooperation against simian immunodeficiency virus replication in rhesus macaques. J Virol 86: 738–745. 22072784 10.1128/JVI.06226-11 PMC3255834

[48] Flores-Villanueva PO, Yunis EJ, Delgado JC, Vittinghoff E, Buchbinder S, et al (2001) Control of HIV-1 viremia and protection from AIDS are associated with HLA-Bw4 homozygosity. Proc Natl Acad Sci USA 98: 5140–5145. 11309482 10.1073/pnas.071548198 PMC33177

[49] Martin MP, Gao X, Lee JH, Nelson GW, Detels R, et al (2002) Epistatic interaction between KIR3DS1 and HLA-B delays the progression to AIDS. Nat Genet 31: 429–434. 12134147 10.1038/ng934

[50] Martin MP, Qi Y, Gao X, Yamada E, Martin JN, et al (2007) Innate partnership of HLA-B and KIR3DL1 subtypes against HIV-1. Nat Genet 39: 733–740. 17496894 10.1038/ng2035 PMC4135476

